# Gene expression profiling and inhibition of adipose tissue accumulation of *G. bimaculatus* extract in rats on high fat diet

**DOI:** 10.1186/s12944-015-0113-3

**Published:** 2015-09-24

**Authors:** Mi Young Ahn, Min-Ji Kim, Ryun Hee Kwon, Jae Sam Hwang, Kun-Koo Park

**Affiliations:** Department of Agricultural Biology, National Academy of Agricultural Science, RDA, Wanju-Gun, 565-851 South Korea; Pharmacogenechips Inc., Chuncheon, 200-160 South Korea

**Keywords:** Anti- atherosclerosis effect, *G. Bimaculatus* extract, Wistar rats, 1-month treatments

## Abstract

**Background:**

Molecular genetic mechanisms underlying the anti-inflammatory effects of ethanol extract (GB) from *G. bimaculatus*, a type of cricket, are not fully elucidated. *G. bimaculatus* was reported to be rich in unsaturated fatty acid and to decrease the omega-6/omega-3 fatty acid ratio when fed to chickens. GB may reduce the amount of fat or increase the unsaturated fatty acid ratio.

**Methods:**

Male Wistar rats fed a high-fat diet (HFD) were orally administered with 5 groups: phosphate buffered saline (PBS, control), GB (100 mg/kg or 200 mg/kg), Pravastatin or *Isaria sinclairii* (IS) extract, which is reported to have fat-reducing effects, for either 1 or 2 months. GB’s sero-biochemial, hematological and anti-oxidizing hepato-cellular biomarker levels were evaluated to dertermine their antilipidemic, anti-inflammatory, and anti-coagulant effect in rats after 1 or 2 month GB treatments on HFD (fat 60 %) Wistar rat. The abdominal and epididymidal fat weight were measured and the composition of fatty acid was analyzed by GC/MS. Microarray analyses were performed with a rat 28 K cDNA clone set array to identify the gene-expression profiles for the GB exposed high fat dieted Wistar rat.

**Results:**

The weight and fatty acid composition of abdominal fat and epididymidal fat, total cholesterol, LDL-cholesterol, and triglyceride in GB treated rats were at lower levels than those of the control group. The anti-oxidant hepato-cellular biomarker levels, protein carbonyl content and malondialdehyde concentration in GB treated rats were significantly decreased. Compared to the control, the GB treated rat group (treated at a dose of 100 and 200 mg/kg), had 190 up-regulated genes including Gpm6a (glycoprotein m6a), Tmem14a (transmembrane protein 14A) and Fasin (fatty acid synthase), with down-regulated 235 genes including Cc121b (chemokine ligand 21b), Glycan1 (glycosylation dependent cell adhesion moleule, Serpinb1a (serine proteinase inhibitor) and Tcrb (T-cell receptor beta chain).

**Conclusion:**

The data suggest Fasin-related fatty acid synthesis and adipose differentiation related protein (Adfp), which is related to obesity, were upregulated by GB treatment, indicating their potential therapeutic markers for anti-atheriosclerosis or inflammation.

## Background

Obesity is a metabolic disorder and the fundamental cause of other fatal diseases including atherosclerosis, hypertension, diabetes, premature aging and cancer [1]. A high fat diet causes diseases such as obesity and changes the DNA gene expression profile [2–4].

**Scheme 1:**
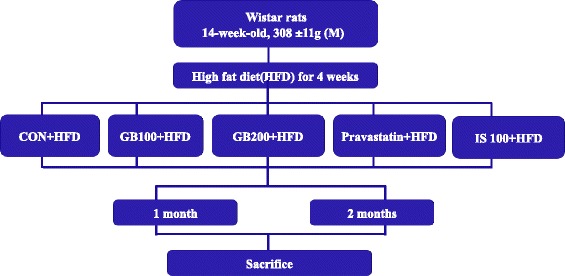
Animal experimental design

Cricket (*Gryllus bimaculatus*) water extract is used in Oriental medicine as a crude drug for treating fever and hypertension, and crickets are currently reared as food for pets [5]. The main components of *G. bimaculatus* are protein (52.81 %), ash (minerals) and fat (21.81 %), including rich essential unsaturated conjugated fatty acids – such as palmitic acid (ω-7, 34.14 %), oleic acid (ω-9, 36.48 %), and linoleic acid (ω-6, 13.58 %) [6]. Water and methanol extracts from crickets were recently found to cause a significant decrease in blood ethanol concentrations by enhancing liver mitochondrial alcohol metabolizing enzymes [7]. The extracts also had protective effects against acute hepatic damage [8].

The fact that *G. bimaculatus* is abundant in unsaturated fatty acid and decrease of omega-6/omega-3 fatty acid ratio when fed to chickens suggest that *G. bimaculatus* may reduce fat or increase unsaturated fatty acid ratio in tissues [6]. The antioxidant effect of GB reported in previous studies may relieve the obese state or obese-related disorders. Recent studies report anti-obesity and anti-diabetic effects are *Isaria sinclairii* (Cicada Dongchunghacho, a fungus cultured on silkworm) [9–11]. Pravastatin (a type of statins), lipid-lowering drug, especially hydroxymethylglutaryl-CoA reductase inhibitor, is widely used in the treatment and prevention of atherosclerotic diseases [12]. Therefore, we assessed the effects of GB compared its antilipidemic activity with *I. sinclarii* ethanol extract or Pravastain as positive controls. In this study, the fatty acid composition in abdominal fat tissue and epididymidal tissue of Wistar rats treated with GB was evaluated and compared to *Isaria sinclaii* [10] extract (IS100) and pravastatin (STA). A high fat diet (HFD) also can cause oxidative stress and was due to lipid peroxidation (malon dialdehyde increase), protein carbonyl content increase, and DNA damage.

We report the sero-biochemical and DNA micro array study of GB in HFD Wistar rats with regard to preventing oxidative stress to proteins, lipids and DNA. This GB holds great promise for use as an anti-obesity drug to decrease fat accumulation in people on high fat diets and prevent changes in liver fat. We demonstrate the potential efficacy of GB in the treatment of anti-lipidemic effect on a HFD rats to be a protective nutraceutical for atherosclerosis disorders, including circulatory disorders, showing gene expression profile with valuable prognostic marker to identify potential therapeutic targets for atherosclerosis and obesity.

## Results

### Clinical sign and food consumption

**Fig. 1:**
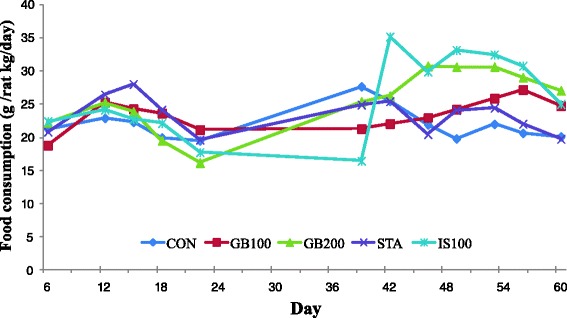
Food consumption changes in Wistar rats treated with GB on a high fat diet

No deaths or adverse clinical signs were apparent due to the ingestion of the *G. bimaculatus* extract or pravastatin. The level of food consumption was similar in all treated groups during the course of the study (Fig. 1). Mean daily food intake was 26.6 g/kg bw/day.

### Body weight and adipose fat weight changes

**Fig. 2:**
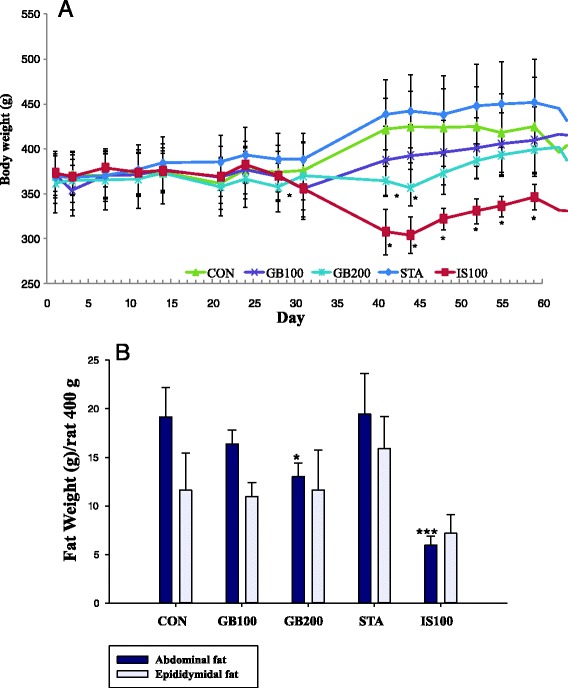
**a** Effect of GB on body weight in high fat (60 %) dieted rat over 2 months. GB100 (200): *G. bimaculatus* ethanol extract 100 (200) mg/kg. STA: Pravastatin 2 mg/kg. IS100: *I. sinclairii* ethanol extract 100 mg/kg. *: significant differences compared with CON group (*p < 0.05). **b** Effect of GB (1 month treatment) on abdominal and epididymidal fat weight on a high fat diet. *: significant differences compared with CON group (*p < 0.05, ***p < 0.001)

There were no toxicologically significant differences in mean body weight between any of the treatment groups (Fig. 2a). During the 1-month administration period, the body weights of the male Wistar rats in the 2 treatment groups were comparable in the control and trexperimental groups. The mean weekly body weights over time are presented in Fig. 2a. However, about 2 months after the experiment, body weight deviation between the groups increased, especially with the IS100 group. The body weight of the IS100 group continued to decrease for some time. The body weight of the IS100 group was significantly different from that of the control group (*p* < 0.05). At sacrifice, the abdominal fat tissues and the epididymidal fat tissues were dissected and weighed to investigate adipose tissue changes. The abdominal fat weight of HFD rats was significantly reduced by *G. bimaculatus* extract (200 mg/kg) (GB200) after 2 months of treatment: Con, 19.16 ± 3.04 g; GB100, 16.37 ± 1.46 (85.4 %); GB 200, 13.03 ± 1.37 g (68.0 %, GB200 vs Con, *p* < 0.05). Figure 2b also shows the declineof abdominal and epididymidal fat weight in HFD rats treated with GB for 1 month. The relative weight of epididymidal fat tissuedecreased to 75.0 % in GB200 after1 month of treatment. The total fat in GB100 and GB200 decreased by 77.9 and 73 % comparedto the CON (Fig. 2b). The fat-reducing effect of GB extract was better than that of pravastatin (STA) in rats.

### Serum biochemical analysis

The changes in serum lipid and lipoprotein profiles after the 1-mo. feeding period are show in Table 1.

**Table 1:** Serological findings for ethanol extract from cricket in Wistar rats on high fat diet over a 1-month treatment period

Item	Unit	CON	GB100	STA	IS100
phospholipid	mg/dL	151.50 ± 17.06	152.00 ± 19.95	178.80 ± 20.20	123.40 ± 10.48
FFA	mEq/L	507.75 ± 45.46	465.75 ± 57.94	532.40 ± 81.22	526.20 ± 126.11
Insulin	mU/ml	<0.2 ± 0.0	<0.2 ± 0.0	<0.2 ± 0.0	<0.2 ± 0.0
Albumin	g/dL	4.05 ± 0.24	3.37 ± 0.21	3.94 ± 0.25	3.48 ± 0.260
T. Bil	mg/dL	0.125 ± 0.05	0.1 ± 0	0.1 ± 0.0	0.14 ± 0.09
ALP	IU/L	133.25 ± 40.46	79.75 ± 13.43	107.50 ± 63.16	168.60 ± 106.11
AST	IU/L	100.5 ± 14.30	143 ± 96.23	116.61 ± 9.36	168.33 ± 25.0
ALT	IU/L	41 ± 6.48	71.25 ± 67.86	49.80 ± 10.23	100.80 ± 80.83
IP	mg/dL	21.48 ± 2.07	14.60 ± 2.32^a^	14.66 ± 1.34	11.14 ± 1.88
yGT	g/dL	<0.3 ± 0.0	<0.3 ± 0.0	<0.3 ± 0.0	<0.3 ± 0.0
CK	IU/L	257.5 ± 79.37	205.50 ± 44.35	377.60 ± 88.71	236.00 ± 93.74
LDH	IU/L	1475.25 ± 311.35	1206.75 ± 375.44	1567.20 ± 344.29	1342.00 ± 344.95
Glucose(S)	mg/dL	340.75 ± 26.48	225.00 ± 49.03	317.00 ± 85.46	213.40 ± 52.18
T. Chol	mg/dL	105.5 ± 7.33	110.25 ± 21.04	117.80 ± 22.40	83.80 ± 12.28
TG	mg/dL	167 ± 33.97	112.75 ± 20.2	151.60 ± 53.35	91.80 ± 40.44
LDL Chol	mg/dL	22.25 ± 4.34	32.25 ± 10.37	24.80 ± 9.20	18.00 ± 6.52
BUN	mg/dL	23.55 ± 1.25	16.2 ± 2.20	22.42 ± 2.27	27.68 ± 5.18
HDL Chol	mg/dL	79 ± 7.75	73.5 ± 7.05	88.20 ± 15.74	63.80 ± 12.99
Creatinine	mg/dL	0.59 ± 0.04	0.501 ± 0.09	0.61 ± 0.09	0.86 ± 0.15
Uric acid	mg/dL	6.18 ± 1.71	5.33 ± 0.22	5.48 ± 1.44	5.88 ± 0.84
Na	nmol/L	127.25 ± 4.65	133.75 ± 5.68	133.40 ± 2.61	133.80 ± 2.78
K	nmol/L	26.78 ± 8.73	20.13 ± 4.02	21.04 ± 1.85	18.86 ± 2.96
Cl	nmol/L	93.5 ± 2.89	95.00 ± 3.16	95.20 ± 2.59	94.40 ± 2.61
T. protein	g/dL	6.75 ± 0.5	6.35 ± 0.42	6.54 ± 0.39	6.38 ± 0.47
CRP(HS)	mg/L	1.68 ± 0.05	0.53 ± 0.25	1.58 ± 0.40	1.16 ± 0.50
Calcium	nmol/L	12.40 ± 0.64	11.90 ± 0.62	12.34 ± 0.87	12.12 ± 0.40

*FFA* free fatty acid, T. *Bil* total bilirubin, *ALP* alkaline phosphatase, *AST(GOT)* glutamate oxaloacetate transaminase, *ALT(GPT)* glutamate pyruvate transaminase, *IP* inorganic phosphorus, *GGT γ*-glutamyl transferase, *CK* creatinine phosphokinase, *LDH* lactate dehydrogenase, *Na* Sodium, *K* potassium, *Cl* chloride, *BUN* blood urea nitrogen, *T. Chol* total cholesterol, *TG* triglyceride, *H. Chol* high cholesterol, *l.Chol* low cholesterol, *Ca* calcium, *CRP* c-reactive protein

Each value represents mean ± S.D. Statistically significant from control (^*^ *P* < 0.05)

CON: PBS (vehicle) treated with murine high fat diet

The data obtained clearly show that high-fat diet ingestion increased the concentration of serum triglyceride and serum triglyceride was decreased in the GB100 experimental rats [control, 167.00 ± 33.97 mg/dL; GB100, 112.75 ± 20.2 mg/dL (GB100 *vs* Con *p* < 0.05); STA, 151.60 ± 53.35 mg/dL]. Serum glucose levels were lower in the GB-treated group than in the control Serum glucose levels were lower in the GB-treated group than in the control (control, 340.75 ± 26.48 mg/dL; GB100, 225.00 ± 49.03 mg/dL (GB100 *vs* Con, *p* < 0.05); Provastastin (STA), 317.00 ± 85.46 mg/dL; IS100, 213.40 ± 52.18 mg/dL). Analysis of C-reactive protein (CRP) levels showed meaningful anti-edema effect with the inhibition of CRP, but the results had no significance (control, 1.68 ± 0.05 mg/L; GB100, 0.53 ± 0.25 mg/L; STA, 1.58 ± 0.40 mg/L; IS100, 1.16 ± 0.5 mg/L). Also, in the sera of GB100-treated rats, free fatty acid (FFA) levels were lower than in the control (control 507.75 ± 4.46 mEq/L; GB100, 465.75 ± 57.094 mEq/L; STA, 532.40 ± 81.22 mEq/L; IS100 526.20 ± 126.11 mEq/L). Triglyceride levels were significantly lower in the GB treated rats compared to the control as follows: control, 167.0 ± 33.97 mg/dL; GB100, 112.75 ± 20.2 mg/dL (GB100 *vs* CON, *p* < 0.05); STA, 151.60 ± 53.35 mg/dL; IS100, 91.80 ± 40.44 mg/dL. The alkaline phosphatase (ALP) levels of the treated groups were lower in the high fat diet fed rats (control, 133.25 ± 40.46; GB100, 79.75 ± 13.43 (control, 133.25 ± 40.46 IU/L; GB100, 79.75 ± 13.43 IU/L (GB100 *vs* CON, *p* < 0.05); STA, 107.50 ± 63.16 IU/L; IS100, 168.60 ± 106.11 IU/L.

The changes in serum lipid and lipoprotein profiles in HFD rats after GB 2 months of treatment are show in Table 2.

**Table 2:** Serological findings for GB in Wistar rats on high fat diet over a 2-month treatment period

Item	Unit	CON	GB100	GB200	STA	IS100
Total protein	g/dL	6.63 ± 0.18	6.75 ± 0.55	6.95 ± 0.18	6.84 ± 0.15	7.00 ± 0.11
Bilirubin	mg/dL	below 0.1	below 0.1	below 0.1	below 0.1	below 0.1
ALP	U/L	38.67 ± 4.73	38.00 ± 4.24	42.75 ± 7.14	39.00 ± 3.16	42.75 ± 8.26
AST	U/L	145.00 ± 28.58	128.50 ± 21.92	144.00 ± 98.18	96.60 ± 18.23	122.50 ± 51.86
ALT	U/L	46.67 ± 1.53	47.50 ± 10.61	81.75 ± 33.31	36.60 ± 13.13	28.50 ± 4.36
GGT	U/L	below 3	below 3	below 3	below 3	below 3
CK	U/L	607.00 ± 188.07	438.00 ± 61.00	676.25 ± 203.71	382.00 ± 36.56	256.00 ± 37.45
LDH	U/L	1577.33 ± 241.14	1830.50 ± 281.50	1897.25 ± 273.26	1179.00 ± 202.53	916.50 ± 93.39
Na	mmol/L	148.50 ± 1.32	151.50 ± 1.50	147.75 ± 1.31	147.40 ± 0.40	145.25 ± 0.85
K	mmol/L	11.20 ± 1.19	9.40 ± 2.40	10.68 ± 1.18	9.24 ± 0.95	10.43 ± 0.81
Cl	mmol/L	97.00 ± 1.08	99.00 ± 3.00	96.75 ± 1.49	98.40 ± 0.87	97.25 ± 1.03
Creatine	mg/dL	0.80 ± 0.03	0.69 ± 0.12	0.79 ± 0.05	0.72 ± 0.03	0.92 ± 0.06
BUN	mg/dL	16.57 ± 0.27	17.00 ± 0.00	19.78 ± 1.29	13.92 ± 0.46	25.73 ± 2.41
Uric acid	mg/dL	3.83 ± 0.48	7.85 ± 0.55	6.53 ± 0.47	6.08 ± 0.56	5.90 ± 0.33
T.Chol	mg/dL	106.00 ± 8.08	110.50 ± 24.50	93.00 ± 3.61	84.40 ± 3.37	100.75 ± 8.00
H.Chol	mg/dL	89.67 ± 6.17	95.50 ± 18.50	79.67 ± 2.40	74.60 ± 2.58	89.25 ± 6.16
L.Chol	mg/dL	21.00 ± 2.00	21.00 ± 17.67	17.67 ± 0.67	15.60 ± 0.81	20.00 ± 2.12
TG	mg/dL	100.50 ± 11.86	100.50 ± 0.50	101.33 ± 20.02	87.20 ± 9.25	54.25 ± 4.01
Glucose	mg/dL	187.67 ± 2.19	163.00 ± 18.00	192.75 ± 67.45	252.00 ± 29.43	198.75 ± 30.38
Ca	mg/dL	11.57 ± 0.07	11.70 ± 0.70	12.05 ± 0.37	12.30 ± 0.42	12.53 ± 0.37
IP	mg/dL	21.10 ± 1.07	19.25 ± 2.25	19.68 ± 1.77	14.44 ± 1.60	11.18 ± 0.85
FFA	uEq/L	907.49 ± 191.70	1071.50 ± 42.50	931.00 ± 81.12	855.00 ± 122.43	738.00 ± 51.22

Each value represents mean ± S.E. statistically significant from control (^*^ *P* < 0.05)

The obtained data clearly show that high-fat diet consumption increased the concentration of serum total cholesterol, TG, and LDL-cholesterol in addition to decreasing the HDL-cholesterol concentrations in the experimental rats after 2 months of treatment. Total cholesterol, LDL- cholesterol, TG, and total lipid in the GB-treated group were lower than in the control group. HDL-cholesterol in the GB-treated group was higher than in the control group.

**Fig. 3:**
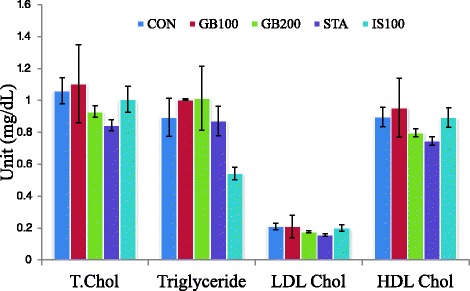
Effect of GB on serum total cholesterol, triglyceride and HDL-cholesterol level in High fat diet rats (2 month GB treatment)

Serum glucose levels were lower in the GB-treated group than in the control (control, 187.67 ± 2.19 mg/dL; GB100, 163.00 ± 18.00 mg/dL; GB200, 192.75 ± 67.45 mg/dL; STA, 252.00 ± 29.43 mg/dL (STA *vs* CON, *p* < 0.05); IS100, 198.75 ± 30.38 mg/dL). In the sera of the GB treated groups, total cholesterol, LDL-cholesterol and triglyceride levels were lower, while HDL-cholesterol was higher than in the control after 2 months. Dose-dependent changes were observed in rats on high-fat diets although there were no significant differences compared with the control group (Fig. 3).

### Hematology and blood chemical analysis

Some dose-dependent changes were observed between the treated and control groups with respect to the hematological parameters at the end of the experiment. An increase in partial thromboplastin time and thrombin time was observed in the treated groups on high fat diets but without significant differences. Minor changes were found in hematological parameters (hematocrit, MCV, MCHC and factor I) for some GB-treated rat groups. However, the effects of GB were not considered adverse because all changes in hematological data including neutrophil, lymphocyte, monocyte and eosinophil were within the normal physiological range. The platelet count of the IS100 group was increased compared to the CON group (*p* < 0.05) (Table 3).

**Table 3:** Hematological findings for extract from cricket in Wistar rats on high fat diet over a 2-month treatment period

	Unit	CON	GB100	GB200	STA	IS100
WBC	10^3^/μl	535 ± 0.54	6.67 ± 1.56	6.28 ± 0.75	4.60 ± 0.35	7.3 ± 0.56
RBC	10μl	8.15 ± 0.09	8.65 ± 0.71	9.04 ± 0.20	8.83 ± 0.19	8.56 ± 0.11
Hgb	g/dL	1496 ± 0.29	15.10 ± 0.50	15.75 ± 0.22	15.78 ± 0.20	14.83 ± 0.09
Hct	%	50.16 ± 1.01	51.50 ± 2.60	53.68 ± 0.88	53.26 ± 0.76	49.65 ± 0.43
MCV	fL	6156 ± 1.15	59.70 ± 1.90	59.43 ± 0.72	60.40 ± 0.76	58.03 ± 0.95
MCH	pg	18.18 ± 0.49	17.55 ± 0.85	17.40 ± 0.36	17.92 ± 0.24	17.30 ± 0.30
MCHC	g/dL	29.84 ± 0.25	29.35 ± 0.45	29.30 ± 0.27	29.64 ± 0.16	29.85 ± 0.14
PLT	10^3^/μl	974.00 ± 67.06	938.50 ± 216.50	1044.00 ± 63.44	954.80 ± 38.77	1240.25 ± 24.32
PTT	sec	98.06 ± 18.58	107.80 ± 36.20	76.03 ± 11.13	74.70 ± 7.26	59.50 ± 12.03
Thrombin time	sec	60.10 ± 13.69	63.40 ± 22.10	53.40 ± 11.06	47.70 ± 6.08	50.90 ± 9.49
Factor I	mg/dL	194.00 ± 3012	216.50 ± 9.50	245.75 ± 18.36	214.60 ± 26.21	247.75 ± 13.03
PT	sce	5.66 ± 0.16	1.59 ± 0.11	1.53 ± 0.04	1.48 ± 0.06	1.46 ± 0.07
Neutrophil	%	21.70 ± 334	13.10 ± 0.60	16.83 ± 2.28	16.72 ± 1.66	18.23 ± 1.89
Lymphocyte	%	75.43 ± UJ oo	85.55 ± 0.55	81.48 ± 2.49	81.22 ± 1.84	79.78 ± 2.08
Monocyte	%	0.90 ± 0.19	0.35 ± 0.05	0.43 ± 0.14	0.64 ± 0.10	0.68 ± 0.19
Eosinophil	%	1.62 ± 0.13	0.85 ± 0.05	1.03 ± 0.13	1.04 ± 0.15	0.88 ± 0.08
Basophil	%	0.34 ± 0.08	0.15 ± 0.05	0.25 ± 0.06	0.38 ± 0.07	158 ± 1.14

*WBC* white blood cell, *RBC* red blood cell, *Hgb* hemoglobin, *Hct* hematocrit, *MCV* mean corpuscular volume, *MCH* mean corpuscular hemoglobin, *MCHC* mean corpuscular hemoglobin concentration, *PLT* partial thromboplastin time, *PT* prothrombin time

CON: PBS (as a vehicle) treated with high fat diet

Each value represents mean ± S.E. Statistically significant from control (P* < 0.05)

### Oxidative protein damage (carbonyl content and catalase) quantitation

**Fig. 4:**
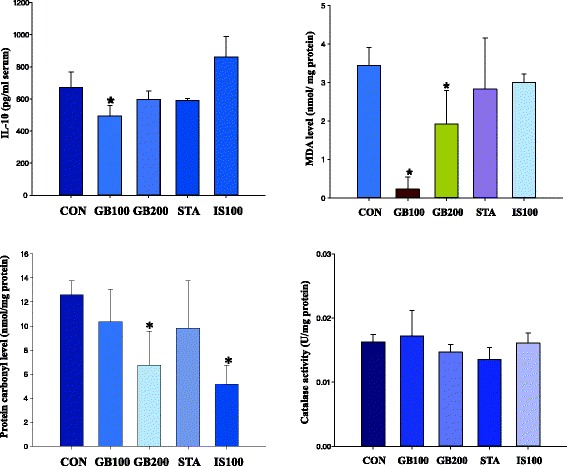
Anti-oxidative effect of GB on lipids (MDA or IL-6) and proteins (carbonyl or catalase content) after 2-month treatment. Each value represents mean ± S.D. statistically significant from control (*P < 0.05)

Protein oxidative stress was evaluated by measuring protein carbonyl content in the blood (Fig. 4). Catalase activity and carbonyl content were assayed as the biomarkers of protein oxidative damage in the high fat dieted rat model.

After 2 months, carbonyl content level was decreased by GB treatment: CON, 12.6 ± 1.1 nmol/mg protein; GB100, 10.4 ± 2.7 nmol/mg protein; GB200 (GB200 *vs* CON, *P* < 0.05), 6.8 ± 2.8 nmol/mg protein; STA, 9.8 ± 4.0 nmol/mg protein; IS100, 5.1 ± 1.6 nmol/mg protein (IS100 *vs* CON, *P* < 0.05). The protein carbonyl content in the blood was reduced in a dose-dependent manner after extract treatments for 2 months. The protein carbonyl concentration was decreased by 82 and 53 % in GB100 and GB200, respectively (Fig. 4).

The catalase activity (Ug/protein) after 2 months of GB treatment was as follows: CON, 0.016 ± 0.001 U/mg protein; GB100, 0.017 ± 0.004 U/mg protein; GB200, 0.014 ± 0.001 U/mg protein; STA (2 mg/kg), 0.014 ± 0.002 U/mg protein; IS100, 0.016 ± 0.002 U/mg protein in hepatocytes (Fig. 4). The catalase activity in all hepatocyte groups was not affected.

### Oxidative lipid damage (malondialdehyde) quantitation

As a lipid oxidative damage marker in lipid oxidative stress states, malondialdehyde level (MDA, nmol/mg/protein) was assayed after 2 months of GB treatment: PBS (CON), 3.44 ± 0.47 nmol/mg/protein; GB 100, 0.23 ± 0.31 (GB100 *vs* CON, *p* < 0.05) nmol/mg/protein, GB 200, 1.92 ± 0.87 nmol/mg/protein (GB200 *vs* CON, *p* < 0.05); STA, 2.83 ± 1.33 nmol/mg/protein; IS100, 3.00 ± 0.22 nmol/mg/protein (Fig. 4). Pravastatin (STA) and IS100 did not affect the lipid peroxidation in hepatocytes.

### Cytokine IL-10 production

Decrease of serum IL-10 level was observed in the GB-treated group. IL-10 activity after 2 months of GB treatment in HFD rat serum was as follows: PBS: 670.5 ± 96.1 pg/ml serum, GB 100: 492.0 ± 66.6 pg/ml serum (GB100 *vs* CON, *p* < 0.05), GB 200: 598.1 ± 51.8 pg/ml serum, STA: 590.7 ± 12.2 pg/ml serum, IS100: 862.4 ± 126.6 pg/ml serum (Fig. 4). STA and IS100 had no statistical differences compared with the CON group.

### DNA microarray

Microarray analysis using a Mouse 28 K cDNA clone set array was performed to identify the gene-expression profiles in the GB treated Wistar rat livers and provided information on potential markers for atherosclerosis. Compared to the control group, the GB treated rats showed 419 (200 mg/kg) and 430 (100 mg/kg) up-regulated genes (15 % increase), and the signal ratio increased from 1.15-fold to 3.28-fold. Gpm6a (glycoprotein m6a), Tmem14a (transmembrane protein 14A) and Fasin (fatty acid synthase) were up-regulated and 421 genes (ratio 0.47 ~ 0.85) including Cc121b (chemokine ligand 21b), Glycan1 (glycosylation dependent cell adhesion moleule, Serpinb1a (serine proteinase inhibitor) and Tcrb (T-cell receptor beta chain) were down-regulated. The data suggests that Fasin related fatty acid synthesis and Adfp, an adipose differentiation related protein associated with obesity, were upregulated by GB treatment. However, a series of genes involved in signal transduction, fatty acid synthesis, energy metabolism (oxidative metabolism) and cellular defenses were more up-regulated, indicating their potential as therapeutic markers for lipid metabolism (Tables 4 and 5).

**Table 4:** Upregulated genes differentially expressed in liver tissue of high fat diet rats treated with *G. bimaculatus* extract over a 1-month period

	G100^a^	G200^b^	Gene title	Gene symbol
1	0.891	1.916	glycoprotein m6a	Gpm6a
2	0.957	1.767	transmembrane protein 14A	Tmem14a
3	1.300	1.416	fatty acid synthase	Fasn
4	0.993	1.396	RT1 class I, locus CE5	RT1-CE5
5	1.370	1.393	ubiquitin-like modifier activating enzyme 5	Uba5
6	0.617	1.372	RT1 class Ib, locus EC2	RT1-EC2
7	1.101	1.358	TWIST neighbor	Twistnb
8	1.303	1.345	coagulation factor C homolog, cochlin	Coch
9	1.497	1.343	X-linked Kx blood group (McLeod syndrome) homolog	Xk
10	1.171	1.341	dedicator of cytokinesis 11	Dock11
11	0.945	1.335	amyloid beta (A4) precursor-like protein 1	Aplp1
12	0.853	1.334	D site of albumin promoter binding protein	Dbp
13	0.794	1.326	RT1 class I, locus CE11-like /// RT1 class I, locus A3	LOC100364500
14	1.860	1.319	Sumo1/sentrin/SMT3 specific peptidase 5	Senp5
15	1.125	1.316	platelet-activating factor acetylhydrolase, isoform 1b	Pafah1b1
16	1.032	1.306	isoprenoid synthase domain containing	Ispd
17	1.163	1.288	osteoglycin	Ogn
18	1.273	1.280	myxovirus (influenza virus) resistance 2	Mx2
19	0.957	1.276	similar to KIAA0802 protein	RGD1308319
20	1.170	1.276	echinoderm microtubule associated protein like 2	Eml2
21	1.061	1.275	Rab40b, member RAS oncogene family	Rab40b
22	1.099	1.268	Complement component 1, q subcomponent-like 3	C1ql3
23	1.399	1.266	family with sequence similarity 135, member A	Fam135a
24	1.377	1.266	Adipose differentiation related protein	Adfp
25	1.091	1.264	acylphosphatase 2, muscle type	Acyp2
26	1.058	1.259	RAD23 homolog B (S. cerevisiae)	Rad23b
27	1.141	1.258	cytochrome P450, family 4, subfamily v, polypeptide 3	Cyp4v3
28	1.113	1.256	Opticin	Optc
29	0.961	1.255	ubiquitin D	Ubd
30	1.038	1.253	nucleosome assembly protein 1-like 3	Nap1l3
31	1.168	1.252	protocadherin alpha 1 /// protocadherin alpha 10	Pcdha1
32	1.183	1.251	similar to CG12279-PA	LOC500420
33	1.058	1.249	solute carrier family 31 (copper transporters), member 1	Slc31a1
34	0.979	1.248	stem-loop binding protein	Slbp
35	1.026	1.242	O-sialoglycoprotein endopeptidase-like 1	Osgepl1
36	1.060	1.240	ring finger protein 141	rnf141
37	1.006	1.240	caveolin 2 /// caveolin 2-like	Cav2
38	1.025	1.240	major facilitator superfamily domain containing 9	Mfsd9
39	0.991	1.238	chemokine (C-X-C motif) ligand 9	Cxcl9
40	1.237	1.237	protein phosphatase 4, regulatory subunit 2	Ppp4r2

^a^GB100/control ratio

^b^GB200/control ratio

**Table 5:** Downregulated genes differentially expressed in liver tissue of high fat diet rats treated with GB over a 1-month period

	GB100^a^	GB200^b^	Gene title	Gene symbol
1	0.478	0.472	chemokine (C-C motif) ligand 21b	Ccl21b
2	0.563	0.563	glycosylation dependent cell adhesion molecule 1	Glycam1
3	0.610	0.583	serine (or cysteine) proteinase inhibitor, clade B, member Serpinb1a	
4	0.625	0.599	T-cell receptor beta chain	Tcrb
5	0.755	0.618	Lymphoid enhancer binding factor 1	Lef1
6	0.615	0.646	T-cell receptor beta chain	Tcrb
7	0.646	0.658	immunoglobulin heavy chain 6	Igh-6
8	0.699	0.658	CD3 molecule, gamma polypeptide	Cd3g
9	0.680	0.680	coronin, actin binding protein 1A	Coro1a
10	0.687	0.687	SATB homeobox 1	Satb1
11	0.623	0.695	similar to RIKEN cDNA A430107P09	LOC100364854
12	0.717	0.701	Fas apoptotic inhibitory molecule 3	Faim3
13	0.703	0.715	lumican	Lum
14	0.924	0.722	interleukin 7 receptor	Il7r
15	0.815	0.731	SATB homeobox 1	Satb1
16	0.869	0.740	CD3 molecule, epsilon polypeptide	Cd3e
17	0.819	0.742	ADP-ribosylation factor-like 5C	Arl5c
18	0.749	0.749	protein tyrosine phosphatase, receptor type, C	Ptprc
19	0.889	0.755	cancer susceptibility candidate 1	Casc1
20	0.879	0.758	LIM domain containing 2	Limd2
21	0.901	0.760	bromodomain containing 4	Brd4
22	1.007	0.764	proenkephalin	Penk
23	0.776	0.765	hypothetical protein LOC100364588	LOC100364588
24	0.915	0.768	phospholipase D1	Pld1
25	0.777	0.769	immunoglobulin joining chain	Igj
26	0.941	0.769	sodium channel, voltage-gated, type IV, alpha subunit	Scn4a
27	0.806	0.770	Zinc finger protein 710	Znf710
28	0.729	0.770	thyroid hormone receptor beta	Thrb
29	0.926	0.774	transition protein 2	Tnp2
30	0.822	0.774	SWI/SNF-related matrix-aa-dependent regulator of c2	LOC685179
31	0.958	0.774	protocadherin gamma subfamily A, 1	Pcdhga1
32	0.821	0.776	tubulin, beta 2c	Tubb2c
33	0.938	0.776	galanin receptor 2	Galr2
34	0.838	0.777	matrix metallopeptidase 12	Mmp12
35	0.777	0.777	SAM and SH3 domain containing 3	Sash3
36	0.771	0.777	TBC1 domain family, member 10C	Tbc1d10c
37	0.981	0.779	2′-5′-oligoadenylate synthetase-like	Oasl
38	0.764	0.779	similar to immunoglobulin light chain variable region	RGD1564318
39	0.812	0.779	POU class 2 associating factor 1	Pou2af1
40	0.783	0.783	CD3 molecule delta polypeptide	Cd3d

^a^GB100/control ratio

^b^GB200/control ratio

### Fatty acid composition in adipose tissue

The fatty acid profile, as indicated GC-MS, showed a slight dose-dependent increase in arachidonic acid (C20: 4n6, AFA) concentration in the epididymidal of male Wistar rats in the GB- treated groups over a 2-month period compared to the control group (Table 6). The high fat dieted Wistar rats treated with GB showed increases in the unsaturated fatty acids (FA) ratio, especially single (mono) FA, but had decreases in saturated fatty acid (Tables 6 and 7).

**Table 6:** Analysis of fatty acid composition in abdominal fat of Wistar rats on high fat diets treated with *G. bimaculatus* extract for 1 month

Comp. of abdominal fat tissue (%)	PBS	GB100	GB200	STA	IS100
Lauric acid (C12:0)	0.04 ± 0.00	0.05 ± 0.00*	0.05 ± 0.00	0.05 ± 0.00*	0.04 ± 0.00
Myristoleic acid (C14:1)	0.02 ± 0.00	0.02 ± 0.00	0.02 ± 0.00*	0.02 ± 0.00	0.02 ± 0.00
Myristic acid (C14:0)	0.72 ± 0.03	0.74 ± 0.04	0.64 ± 0.13	0.66 ± 0.02	0.69 ± 0.01
Pentadecanoic acid (C15:0)	0.12 ± 0.00	0.12 ± 0.00	0.24 ± 0.12	0.12 ± 0.00	0.12 ± 0.01
Palmitoleic acid (C16:1)	1.37 ± 0.14	1.52 ± 0.22	1.76 ± 0.07*	1.18 ± 0.17	1.31 ± 0.04
Palmitic acid (C16:0)	21.49 ± 0.49	21.42 ± 0.59	21.46 ± 0.39	21.20 ± 0.14	20.85 ± 0.17
Heptadecanoic acid (C17:0)	0.29 ± 0.01	0.28 ± 0.01	0.29 ± 0.01	0.33 ± 0.01*	0.30 ± 0.01
Linoleic acid (C18:2)	34.37 ± 2.80	35.24 ± 0.91	35.50 ± 0.27	31.56 ± 0.92	34.70 ± 0.90
Oleic acid (C18:1)	33.56 ± 2.35	32.94 ± 0.89	32.33 ± 0.57	35.84 ± 0.88	33.79 ± 0.89
Stearic acid (C18:0)	6.92 ± 0.13	6.72 ± 0.37	6.75 ± 0.17	7.90 ± 0.21*	7.09 ± 0.20
Arachidonic acid (C20:4)	0.20 ± 0.06	0.14 ± 0.02	0.13 ± 0.01	0.19 ± 0.00	0.19 ± 0.04
Eicosapentaenoic acid (C20:5)	0.02 ± 0.00	0.02 ± 0.00	0.02 ± 0.00	0.03 ± 0.00	0.01 ± 0.00
Eicosatrienoic acid (C20:3)	0.06 ± 0.01	0.05 ± 0.01	0.05 ± 0.00	0.06 ± 0.00	0.06 ± 0.01
Eicosadienoic acid (C20:2)	0.25 ± 0.00	0.25 ± 0.03	0.24 ± 0.01	0.29 ± 0.00*	0.24 ± 0.01
Eicosenoic acid (C20:1)	0.37 ± 0.01	0.38 ± 0.04	0.39 ± 0.01	0.43 ± 0.01*	0.41 ± 0.01
Eicosanoic acid (C20:0)	0.09 ± 0.01	0.09 ± 0.01	0.09 ± 0.01	0.10 ± 0.00	0.10 ± 0.01
Docosahexaenoic acid (C22:6)	0.11 ± 0.08	0.04 ± 0.00	0.04 ± 0.00	0.04 ± 0.01	0.09 ± 0.04
Saturated fatty acid	29.66 ± 0.59	29.41 ± 0.39	29.52 ± 0.40	30.36 ± 0.18	29.19 ± 0.28
Unsaturated fatty acid	70.34 ± 0.59	70.59 ± 0.39	70.48 ± 0.40	69.64 ± 0.18	70.81 ± 0.28
Single unsaturated fatty acid	35.33 ± 1.06	34.86 ± 1.22	34.50 ± 2.53	37.47 ± 2.23	35.52 ± 0.42
Poly unsaturated fatty acid	35.02 ± 2.73	35.73 ± 0.88	35.98 ± 0.29	32.16 ± 0.93	35.29 ± 0.93
Comp. of epididymidal fat tissue (%)	PBS	GB100	GB200	STA	IS100
Lauric acid (C12:0)	0.04 ± 0.00	0.04 ± 0.00	0.04 ± 0.00	0.05 ± 0.00	0.04 ± 0.00
Myristoleic acid (C14:1)	0.02 ± 0.00	0.03 ± 0.00	0.03 ± 0.00	0.03 ± 0.00	0.03 ± 0.00
Myristic acid (C14:0)	0.70 ± 0.04	0.78 ± 0.02	0.74 ± 0.01	0.75 ± 0.01	0.73 ± 0.03
Pentadecanoic acid (C15:0)	0.12 ± 0.01	0.14 ± 0.01	0.14 ± 0.00*	0.13 ± 0.00	0.14 ± 0.01
Palmitoleic acid (C16:1)	1.66 ± 0.15	1.77 ± 0.07	1.83 ± 0.05	1.76 ± 0.06	1.73 ± 0.11
Palmitic acid (C16:0)	21.89 ± 0.40	21.82 ± 0.17	21.95 ± 0.21	20.66 ± 0.20*	20.73 ± 0.12
Heptadecanoic acid (C17:0)	0.26 ± 0.01	0.27 ± 0.01	0.27 ± 0.00	0.28 ± 0.01	0.28 ± 0.01
Linoleic acid (C18:2)	36.05 ± 0.96	38.21 ± 1.19	37.86 ± 0.60	39.68 ± 1.24*	38.29 ± 0.08
Oleic acid (C18:1)	32.41 ± 0.65	30.19 ± 1.23	30.69 ± 0.52	29.48 ± 1.00*	31.27 ± 0.27
Stearic acid (C18:0)	5.94 ± 0.07	5.87 ± 0.09	5.57 ± 0.07*	6.14 ± 0.13	5.75 ± 0.03
Arachidonic acid (C20:4)	0.16 ± 0.01	0.13 ± 0.03	0.15 ± 0.00	0.21 ± 0.03	0.19 ± 0.01
Eicosapentaenoic acid (C20:5)	0.01 ± 0.01	0.02 ± 0.01	0.03 ± 0.00*	0.03 ± 0.00*	0.03 ± 0.00
Eicosatrienoic acid (C20:3)	0.06 ± 0.00	0.06 ± 0.00	0.05 ± 0.00	0.07 ± 0.00	0.07 ± 0.01
Eicosadienoic acid (C20:2)	0.25 ± 0.01	0.25 ± 0.01	0.23 ± 0.01	0.28 ± 0.01	0.27 ± 0.02
Eicosenoic acid (C20:1)	0.33 ± 0.01	0.32 ± 0.01	0.30 ± 0.00*	0.34 ± 0.01	0.33 ± 0.01
Eicosanoic acid (C20:0)	0.06 ± 0.00	0.06 ± 0.00	0.06 ± 0.00	0.06 ± 0.00	0.05 ± 0.00
Docosahexaenoic acid (C22:6)	0.03 ± 0.00	0.04 ± 0.00	0.06 ± 0.00*	0.06 ± 0.02	0.07 ± 0.00*
Saturated fatty acid	29.01 ± 0.38	28.98 ± 0.18	28.76 ± 0.16	28.06 ± 0.32	27.71 ± 0.16
Unsaturated fatty acid	70.99 ± 0.38	71.02 ± 0.18	71.24 ± 0.16	71.94 ± 0.32	72.29 ± 0.16
Single unsaturated fatty acid	34.42 ± 0.71	32.32 ± 1.26	32.86 ± 0.54	31.61 ± 0.97	33.36 ± 0.15
Poly unsaturated fatty acid	36.57 ± 0.97	38.71 ± 1.15	38.38 ± 0.59	40.33 ± 1.29	38.93 ± 0.05

Each value represents mean ± SE

Asterisk marks (*) mean significant differences compared with control (PBS) group (*p* < 0.05)

**Table 7:** Analysis of fatty acid composition in abdominal fat of Wistar rats on a high fat diet treated with *G. bimaculatus* extract for 2 months

Comp. of abdomianlfat tissue (%)	PBS	GB100	GB200	STA	IS100
Lauric acid (C12:0)	0.04 ± 0.00	0.05 ± 0.00	0.05 ± 0.00	0.05 ± 0.01	0.06 ± 0.01
Myristoleic acid (C14:1)	0.02 ± 0.00	0.02 ± 0.00	0.02 ± 0.00	0.02 ± 0.00	0.02 ± 0.00
Myristic acid (C14:0)	0.78 ± 0.04	0.77 ± 0.08	0.81 ± 0.06	0.78 ± 0.01	0.88 ± 0.08
Pentadecanoic acid (C15:0)	0.13 ± 0.00	0.14 ± 0.00*	0.14 ± 0.02	0.15 ± 0.01	0.15 ± 0.00*
Palmitoleic acid (C16:1)	1.74 ± 0.33	1.98 ± 0.22	1.93 ± 0.12	2.08 ± 0.20	1.86 ± 0.23
Palmitic acid (C16:0)	19.01 ± 0.57	18.85 ± 0.61	19.85 ± 1.05	18.25 ± 0.94	19.57 ± 1.27
Heptadecanoic acid (C17:0)	0.35 ± 0.04	0.40 ± 0.03	0.46 ± 0.03	0.37 ± 0.03	0.44 ± 0.03
Linoleic acid (C18:2)	36.15 ± 1.91	40.11 ± 2.23	37.26 ± 6.88	40.18 ± 0.89	38.90 ± 1.91
Oleic acid (C18:1)	32.10 ± 2.20	28.12 ± 0.80	28.54 ± 5.66	28.88 ± 1.53	26.16 ± 1.08
Stearic acid (C18:0)	8.29 ± 0.45	8.35 ± 0.69	9.28 ± 0.33	7.86 ± 0.69	10.16 ± 0.38*
Arachidonic acid (C20:4)	0.20 ± 0.04	0.18 ± 0.05	0.24 ± 0.02	0.24 ± 0.04	0.32 ± 0.07
Eicosapentaenoic acid (C20:5)	0.04 ± 0.01	0.03 ± 0.00	0.04 ± 0.00	0.03 ± 0.00	0.04 ± 0.01
Eicosatrienoic acid (C20:3)	0.08 ± 0.01	0.06 ± 0.02	0.08 ± 0.01	0.08 ± 0.01	0.09 ± 0.02
Eicosadienoic acid (C20:2)	0.35 ± 0.02	0.30 ± 0.07	0.43 ± 0.03	0.35 ± 0.02	0.48 ± 0.11
Eicosenoic acid (C20:1)	0.56 ± 0.02	0.49 ± 0.09	0.65 ± 0.05	0.54 ± 0.01	0.69 ± 0.11
Eicosanoic acid (C20:0)	0.12 ± 0.01	0.11 ± 0.01	0.15 ± 0.01	0.12 ± 0.02	0.15 ± 0.02
Docosahexaenoic acid (C22:6)	0.04 ± 0.01	0.04 ± 0.00	0.07 ± 0.02	0.04 ± 0.00	0.06 ± 0.00
Saturated fatty acid	28.73 ± 0.09	28.67 ± 1.43	30.74 ± 1.16	27.57 ± 1.67	31.39 ± 1.70
Unsaturated fatty acid	71.27 ± 0.09	71.33 ± 1.43	69.26 ± 1.16	72.43 ± 1.67	68.61 ± 1.70
Single unsaturated fatty acid	34.42 ± 1.92	30.60 ± 0.67	31.13 ± 5.71	31.51 ± 1.71	28.73 ± 0.94
Poly unsaturated fatty acid	36.84 ± 1.87	40.73 ± 2.10	38.13 ± 6.86	40.92 ± 0.96	39.88 ± 1.71
Comp. of epididymidal fat tissue (%)	PBS	GB100	GB200	STA	IS100
Lauric acid (C12:0)	0.06 ± 0.01	0.06 ± 0.00	0.07 ± 0.01	0.06 ± 0.00	0.09 ± 0.02
Myristoleic acid (C14:1)	0.04 ± 0.00	0.05 ± 0.01	0.04 ± 0.01	0.03 ± 0.01	0.06 ± 0.02
Myristic acid (C14:0)	1.00 ± 0.10	0.90 ± 0.04	0.97 ± 0.06	0.89 ± 0.06	1.03 ± 0.03
Pentadecanoic acid (C15:0)	0.17 ± 0.01	0.19 ± 0.01	0.19 ± 0.01	0.16 ± 0.01	0.19 ± 0.01
Palmitoleic acid (C16:1)	2.44 ± 0.29	3.18 ± 0.92	2.61 ± 0.35	3.06 ± 0.31	2.57 ± 0.47
Palmitic acid (C16:0)	18.63 ± 0.27	18.43 ± 1.41	19.53 ± 0.38	19.97 ± 0.58	18.71 ± 0.72
Heptadecanoic acid (C17:0)	0.36 ± 0.04	0.38 ± 0.05	0.41 ± 0.02	0.39 ± 0.02	0.38 ± 0.03
Linoleic acid (C18:2)	39.26 ± 1.15	39.86 ± 0.98	36.49 ± 2.13	36.59 ± 1.16	34.77 ± 3.41
Oleic acid (C18:1)	28.85 ± 1.57	27.56 ± 0.96	29.85 ± 1.81	29.92 ± 1.35	32.79 ± 3.43
Stearic acid (C18:0)	7.50 ± 0.22	6.96 ± 1.47	7.71 ± 0.29	7.06 ± 0.41	7.44 ± 0.72
Arachidonic acid (C20:4)	0.32 ± 0.02	0.56 ± 0.06*	0.48 ± 0.07	0.40 ± 0.08	0.47 ± 0.07
Eicosapentaenoic acid (C20:5)	0.05 ± 0.01	0.08 ± 0.03	0.07 ± 0.02	0.05 ± 0.02	0.05 ± 0.02
Eicosatrienoic acid (C20:3)	0.11 ± 0.01	0.17 ± 0.02*	0.15 ± 0.02	0.14 ± 0.02	0.15 ± 0.02
Eicosadienoic acid (C20:2)	0.46 ± 0.01	0.51 ± 0.01	0.52 ± 0.04	0.51 ± 0.02	0.46 ± 0.06
Eicosenoic acid (C20:1)	0.56 ± 0.02	0.62 ± 0.07	0.58 ± 0.02	0.58 ± 0.03	0.54 ± 0.09
Eicosanoic acid (C20:0)	0.09 ± 0.01	0.09 ± 0.03	0.08 ± 0.00	0.09 ± 0.01	0.12 ± 0.01*
Docosahexaenoic acid (C22:6)	0.10 ± 0.01	0.37 ± 0.08*	0.24 ± 0.05	0.11 ± 0.04	0.19 ± 0.06
Saturated fatty acid	27.80 ± 0.33	27.03 ± 3.00	28.96 ± 0.66	28.61 ± 0.91	27.96 ± 1.19
Unsaturated fatty acid	72.20 ± 0.33	72.97 ± 3.00	71.04 ± 0.66	71.39 ± 0.91	72.04 ± 1.19
Single unsaturated fatty acid	31.89 ± 1.39	31.41 ± 1.81	33.09 ± 1.66	33.59 ± 1.35	35.96 ± 3.81
Poly unsaturated fatty acid	40.31 ± 1.10	41.57 ± 1.19	37.95 ± 2.14	37.80 ± 1.24	36.08 ± 3.37

Each value represents mean ± SE

Asterisk marks (*) mean significant differences compared with control (PBS) group (*p* < 0.05)

In abdominal GB100 or GB200 fat tissue of 1-month treatment period, mono (single) unsaturated fatty acid composition was decreased whereas poly unsaturated fatty acid composition was increased with dose-dependent manner (each group *vs* CON, *p* < 0.05). In epididymidal fat tissue of GB100 or GB200 HFD rats of 1-month treatment period, the composition of docosahexaenoic acid in epdidymidal fat tissue showed dose-dependent decrease with significant difference (Gb200 *vs* CON, *p* < 0.05). In 1-month treatment groups, eicosapentaenoic acid and docosahexaenoic acid composition were increased with statistical significance (GB100 *vs* CON, *p* < 0.05; STA *vs* CON, *p* < 0.05).

In abdominal fat of 2-month treated HFD rats, poly unsaturated fatty acid were increased, and monounsaturated fatty acid were dose-dependently decreased (each group *vs* CON, *p* < 0.05). Stearic acid composition in IS100 group was higher compared with control group (IS100 *vs* CON, *p* < 0.05).

In epididymidal fat of 2-month treated rats, arachidonic acid and docosahexaenoic acid composition in GB100 (GB100 *vs* CON, *p* < 0.05), and eicosanoic acid composition in IS100 showed significant differences compared with control (IS100 *vs* CON, *p* < 0.05).

## Discussion

A high-fat diet (HFD) induces obesity and obesity related metabolic complications such as adipose inflammation, hepatic steatosis and hyperlipidemia [13]. Among various animal sources tested [14], cricket extract was found to be a potent functional food; the adipose tissue fat weight of Wistar rats treated with GB over a 2-month period was decreased, especially the abdominal fat and epididymidal fat. However, body weight was not significantly different in the GB treated and control groups.

Modulating inflammation in adipose tissue ameliorates obesity-associated metabolic complications [15]. GB extract slightly lowered blood pressure and creatinine phosphokinase or otherwise increased thrombin time, showing anticoagulant activity. Repair of protein oxidative damage caused by a high fat diet, carbonyl content and lipid oxidative damage (malondialdehyde, MDA) were decreased by GB treatment [16]. Hepatic mRNA expression of IL-10 was reported to be increased in obese C57BL6/J mice on high-fat diets [17] and overexpression of IL-10 prevented weight gain in animals on HFD [18].

A high-fat diet decreases energy expenditure and the expression of genes controlling lipid metabolism, mitochondrial function and skeletal system development in the adipose tissue, along with increased expression of extracellular matrix remodeling- and inflammation-related genes [19].

This study observed meaningful gene expression profiles. Compared to the control, the GB extract-treated rat group (at a dose of 100 and 200 mg/kg) had 190 up-regulated genes including Gpm6a (glycoprotein m6a) [20], Tmem14a (transmembrane protein 14A) [21] and Fasin (fatty acid synthase) [22] (Table 3) and 235 down-regulated genes including Cc121b (chemokine ligand 21b), Glycan1 (glycosylation dependent cell adhesion molecule [23], Serpinb1a (serine proteinase inhibitor) [24] and Tcrb (T-cell receptor beta chain) [16] (Table 4).

In accordance with the decreased weight of abdominal fat tissue, total cholesterol, LDL-cholesterol, and triglyceride in GB treated rats were lower than those of the control (PBS treated) group. In the sera of rats treated with GB for 1-month, free fatty acid levels were reduced in a dose-dependent manner. In addition, glucose, triglyceride and alkine phosphatase levels were also decreased in the 100 mg/kg GB extract-treated group. The composition of saturated fatty acid was decreased, while unsaturated fatty acid and poly unsaturated fatty acid were increased in the epididymal adipose tissue, suggesting that a diet rich in poly-unsaturated fatty acid decreases adipose tissue mass and suppresses the development of obesity in rats [3, 25].

## Conclusions

GB demonstrated anti-lipidemic effects in Wistar high fat dieted rats by significantly reducing serum triglyceride and alkaline phosphatase levels. Consequently, GB may be a protective nutraceutical for atherosclerosis disorders, including circulatory disorders. The gene expression profile of high fat dieted Wistar rats treated with GB is a valuable prognostic marker that can be used to identify potential therapeutic targets for atherosclerosis.

## Materials and methods

### Materials

*G. bimaculatus* was reared in a cricket farm located in Jungsun, Kangwon-Do, South Korea. The cricket was freeze-dried at the Department of Agricultural Biology, National Academy of Agricultural Science, Korea.

### Preparation of G. bimaculatus extract (GB)

Dried *G. bimaculatus* (1 kg) was homogenized and soaked then extracted three times with 70 % ethanol by ultrasonification for 30 min. The samples were filtered through Whatman filter paper and concentrated by evaporation and freeze-drying. The dried powder (ethanol extract, GB) was dissolved in saline prior to use in the test solution. *Isaria sinclairii* was also extracted with 70 % ethanol through sonication, evaporation and freeze-drying using the same method as for GB.

### Animals

Han Tac Sam-WH (Wistar) rats (male), weighing 308.0 ± 11.0 g at 14-weeks of age, were obtained from Samtako Co. Ltd. (Osan, Korea) and divided into five groups of ten rats including the Wistar control group. All procedures were in accordance with the NIH Guidelines for the Care and Use of Laboratory Animals. All experiments were approved by the Laboratory Animals’ Ethical Committee of the National Academy of Agricultural Science, RDA and followed national guidelines for the care and use of animals (individual housing). The rats were acclimated for four weeks under normal husbandry conditions (23 ± 2 °C, 55 ± 10 %, humidity and 12 h light/dark cycle) and fed a high (60 %) fat diet, D12492 (Research Diet Inc., USA) and water *ad libitum*. The rats were distributed into the following 5 groups (*n* = 10) with similar weights: I: control group, PBS treatment, II: treated with 100 mg/kg *G. bimaculatus* ethanol extract, III: treated with 200 mg/kg *G. bimaculatus* ethanol extract, IV: treated with 2 mg/kg Pravastatin (CJ Heathcare CO., Korea), V: 100 mg/kg *Isaria sinclairii* ethanol extract. Each group (5 sample groups) was maintained for a one month or two month- period (Scheme 1).

### Organ weights

About five groups named CON, GB100, GB200, STA, IS100, divided 1 month- or 2 month- period subgroup (*n* = 5). The absolute and relative (organ-to-body weight ratios) weights of the following organs were measured: adrenal glands, kidneys, heart, liver, lung, spleen, stomach, pancreas, thymus and ovaries.

### Blood sampling and blood, plasma, serum assay

On five groups named CON, GB100, GB200, STA, IS100, divided 1 month- or 2 month-period subgroup (*n* = 5), after 1 month of treatment, blood (~3 ml) was collected from the posterior vena cava under light CO_2_ inhalation and used for serum chemistry measurements. The parameters examined included total protein, total bilirubin, glucose, glutamic pyruvic transaminase (GPT), glutamic oxaloacetic transaminase (GOT), γ-glutamyl transferase (GGT), alkaline phosphatase (ALP), CK(creatinine phosphokinase), lactic dehydrogenase (LDH), total cholesterol, HDL cholesterol, LDL cholesterol, blood urea nitrogen (BUN), creatinine, triglyceride, uric acid, sodium, potassium and chloride. All parameters were evaluated using an autoanalyzer (Hitachi 7060 automatic clinical analyzer, Tokyo).

### Liver homogenate preparation for oxidative damage detection

About five groups named CON, GB100, GB200, STA, IS100, divided 1 month- or 2 month- period subgroup (*n* = 5), liver tissues were homogenized on ice in a 10-fold volume lysis buffer PRO-PREP™ Protein extraction solution (*i*NtRON, Busan, Korea). The supernatant of the liver homogenate after centrifugation (800 g, 10 min) was assayed for carbonyl content or catalase activity.

### Oxidative protein damage (carbonyl content and catalase) quantitation

On the supernatant of liver homogenate and blood of five groups named CON, GB100, GB200, STA, IS100, divided 1 month- or 2 month- period subgroup (*n* = 3), the carbonyl content was determined with an enzyme-linked immunoassay according to the manufacturer’s protocol for the OxiSelect™ protein carbonyl ELISA kit, Cell Biolabs, Inc. (Sandiego, USA). Catalase activity (CAT, U/mg protein) was measured according to the method based on CAT-mediated decomposition of H_2_O_2_. Potassium phosphate buffer (50 mM, pH 7.0, 0.9 ml) was added to 0.1 ml of the sample followed by H_2_O_2_ solution (1 ml, 30 mM). The decrease in the absorbance at 240 nm was measured for 90 s [26].

### Oxidative lipid damage (malondialdehyde) quantitation

On five groups named CON, GB100, GB200, STA, IS100, divided 1 month- or 2 month- period subgroup (*n* = 3), to determine the oxidative lipid damage in GB treated rat hepatocytes, malondialdehyde (MDA) levels were measured with a lipid peroxidation assay using the color method involving thiobarbituric acid reactive substances (TBARS) at 535 nm. The liver homogenate (0.5 ml) mentioned earlier as well as sodium dodecyl sulfate (7 % SDS, 1 ml) was incubated for 30 min at 37 °C before mixing with TBA (0.67 %, 2 ml, 1: 1 with acetic acid) and adding to tubes. The tubes were mixed, placed in boiling water (100 °C) for 50 min then mixed with butanol (5 ml). 1,1,3,3-tetraethoxypropane was used as a standard [27].

### Cytokine IL-10 assay: R&D kit

On five groups named CON, GB100, GB200, STA, IS100, divided 1 month- or 2 month- period subgroup (*n* = 3), the IL-10 level in GB extract-treated rat serumwas measured using commercial ELISA kits (Quantikine, R&D Systems, Inc., Minneapolis, MN, USA) according to the manufacturer’s instructions.

### DNA microarray procedure

After histopathology analysis, microarray hybridization was performed on liver samples (Control, GB 100 and GB200). The total RNA was isolated from the liver using a Qiagen RNeasy Midi Kit (Qiagen, Valencia, USA). A regular microarray was carried out according to the manufacturer’s instructions for the FairPlay™ microarray labeling kit (Stratagene, La Jolla, CA). Briefly, 20 μg total RNA from the liver was reverse-transcribed into single stranded cDNA. The cDNA was purified with ethanol precipitation and resuspended in 5 μl of 2x coupling buffer, then coupled with 5 μl of Cy3 or Cy5 dye for 1 h in the dark. The labeled control liver cDNA and the treated liver cDNA were combined and purified. The labeled cDNA was mixed with 1.5 μl of 10 μg/μl salmon DNA, 1.5 μl of 8 μg/μl poly d(A), 1.5 μl of 4 μg/μl yeast tRNA, 4.5 μl of 20x SSC and 0.75 μl of 10 % SDS, heated at 99 °C for 2 min, and incubated at 45 °C for 15 min. The labeled DNA was loaded onto a microarray chip. A hybridization chamber was assembled with the microarray chip and submerged in a water bath overnight at 60 °C. The microarray chip was washed in wash buffer I (2x SSC, 0.1 % SDS) for 15 min, then in wash buffer II (1x SSC) for 5 min and in wash buffer III (0.2x SSC) for 15 min. The slide was dried by centrifuging at 500 g for 15 min and scanned with a BMS Array Scanner, applied precision Array WoRx eBiochip Reader (BioRad, Dallas, USA) using the Cy3 and Cy5 channels [28].

### Analysis of fatty acid composition in rat adipose tissue

On five groups named CON, GB100, GB200, STA, IS100, divided 1 month- or 2 month- period subgroup (*n* = 4), for epididymidal and abdominal fat analysis, the concentrations of free fatty acids and fatty acid composition were analyzed for 29 fatty acids in adipose tissue using gas chromatography-mass spectroscopy (GC-MS). Each cut adipose or epididymidal tissue (0.1 g) was collected and extracted overnight bwith a chloroform: methanol (2:1) mixture. The filtered solution was removed under nitrogen gas. The lipids were then saponified by alkaline hydrolysis of phospholipids at 100 °C with 0.5 N methanolic sodium hydroxide and methylated at 100 °C with 14 % BF_3_ for 15 min. The top layer was transferred to petroleum ether and analyzed by GC/MS (Aglient 6890GC, Aglient 5973 N mass detector, EI mode) with a HP-5 capillary column (Aglient Technolgies, Palto alto, Ca, USA). The inlet temperature was 250 °C and the MS transfer line was kept constant at 230 °C. The oven temperature was held at 180 °C for 20 min, then programmed at 10 °C/min to 230 °C and held for 10 min. Quantification was achieved using a mixed 37 fatty acid standard: Sigma L9405, 10 ρg/mL (Sigma-Aldrich Inc.). Linoleic acid (C18:2n6) was used as an internal standard.

### Statistical analysis

The means and standard error of all parameters studied were determined for each group using the ANOVA test. A Student’s *t*-test was carried out to determine significant differences between control and treated groups. A *p* value <0.05 was considered significant.

## Abbreviations

ALP: Alkaline phosphatase
ALT(GPT): Glutamate pyruvate transaminase
AST(GOT): Glutamate oxaloacetate transaminase
BUN: Blood urea nitrogen
Ca: Calcium
CK: Creatinine phosphokinase
Cl: Chloride
CRP: C-reactive protein
FFA: Free fatty acid
GB: *Gryllus bimaculatus* ethanol extract
GB100: *Gryllus bimaculatus* ethanol extract 100 mg/kg
GB200: *Gryllus bimaculatus* ethanol extract 200 mg/kg
GGT: γ-glutamyl transferase
H. Chol: High cholesterol
HFD: High-fat diet
Hct: Hematocrit
Hgb: Hemoglobin
l.Chol: Low cholesterol
IP: Inorganic phosphorus
IS100: *I. sinclairii* ethanol extract 100 mg/kg
LDH: Lactate dehydrogenase
MCH: Mean corpuscular hemoglobin
MCHC: Mean corpuscular hemoglobin concentration
MCV: Mean corpuscular volume
K: Potassium
Na: Sodium
T. Chol: Total cholesterol
TG: Triglyceride
PLT: Partial thromboplastin time
STA: Provastastin
PT: Prothrombin time
RBC: Red blood cell
T. Bil: Total bilirubin
WBC: White blood cell
